# Valoración bioquímica en la enfermedad hepática grasa asociada a la disfunción metabólica

**DOI:** 10.1515/almed-2020-0062

**Published:** 2021-02-05

**Authors:** Armando R. Guerra-Ruiz, Gregori Casals, Paula Iruzubieta, Marta Lalana, Alba Leis, Rosa María López, Javier Crespo, Manuel Morales-Ruiz

**Affiliations:** Grupo de trabajo de valoración bioquímica de la enfermedad hepática, Sociedad Española de Medicina de Laboratorio (SEQC-ML), Barcelona, España; Sociedad Española de Patología Digestiva (SEPD), Madrid, España; Servicio de Análisis Clínicos, Hospital Universitario Marqués de Valdecilla, Santander, España; Servicio de Bioquímica y Genética Molecular, CDB, Hospital Clínic de Barcelona, IDIBAPS, CIBEREHD, Barcelona, España; Servicio Aparato Digestivo, Hospital Universitario Marqués de Valdecilla, Grupo de Investigación Clínica y Traslacional en Enfermedades Digestivas, IDIVAL, Santander, España; Servicio de Análisis Clínicos, Hospital de Barbastro, Huesca, España; Servicio de Análisis Clínicos y Bioquímica, Laboratori Clínic Metropolitana Nord, Hospital Universitari Germans Trias i Pujol, Badalona, España; Unidad de Patología hepática, Departamentos de Bioquímica y Microbiología, Hospital Universitari Vall d’Hebron, Universitat Autònoma de Barcelona, Barcelona, España; Servicio de Bioquímica y Genética Molecular-Hospital Clínic de Barcelona, IDIBAPS, CIBERehd, Departamento de Biomedicina de la Facultad de Medicina y Ciencias de la Salud-Universidad de Barcelona, Barcelona, España

**Keywords:** enfermedad hepática grasa asociada a disfunción metabólica, esteatohepatitis, esteatosis hepática, fibrosis hepática, marcadores séricos

## Abstract

La enfermedad hepática grasa asociada a la disfunción metabólica (MAFLD) se define por el acúmulo de grasa en el hígado en presencia de alteraciones metabólicas. Suele cursar de forma asintomática y puede progresar a formas graves de enfermedad hepática, ligadas a la aparición de inflamación y/o fibrosis. Su prevalencia es muy elevada (26%), resultando en un alto número de pacientes con riesgo de presentar una enfermedad hepática avanzada. El presente documento describe los marcadores serológicos más relevantes en la caracterización y diagnóstico de la MAFLD, y se propone un ejemplo de su integración en un algoritmo diagnóstico en práctica clínica habitual. En la actualidad se dispone de índices serológicos útiles en el manejo de los pacientes con MAFLD, especialmente en la estratificación del riesgo de la presencia fibrosis. Una gran parte de la población está en riesgo de desarrollar enfermedad hepática grave. La integración de los marcadores serológicos no invasivos en la estratificación del riesgo de fibrosis hepática puede contribuir a un mejor control y manejo de los pacientes con MAFLD.

## Definición e introducción

La enfermedad del hígado graso asociada a la disfunción metabólica (MAFLD en sus siglas en inglés, Metabolic (disfunction) Associated Fatty Liver Disease), anteriormente denominada enfermedad del hígado graso no alcohólico (NAFLD, en sus siglas en inglés) [1] engloba un amplio espectro de lesiones hepáticas cuyo denominador común es la presencia de un acúmulo de grasa en el hígado, conocido como esteatosis. Hasta el momento había sido necesaria la exclusión del consumo significativo de alcohol y ciertos fármacos para establecer el diagnóstico de la NAFLD. Sin embargo, la nueva definición de MAFLD elimina los criterios de exclusión e introduce criterios “positivos” en el diagnóstico de esta entidad. De esta forma, el diagnóstico de MAFLD [2] se basa en el hallazgo, ya sea histológico (biopsia), por técnicas de imagen, o por marcadores séricos, de acumulación de grasa hepática (presencia de esteatosis) que se acompaña de uno de los siguientes criterios: sobrepeso u obesidad, diabetes mellitus tipo 2, y/o evidencias de alteraciones metabólicas. Estas últimas son comunes a la actual definición de síndrome metabólico, y las presentamos en la Tabla 1.

**Tabla 1:** Evidencia de alteraciones metabólicas definido como la presencia de al menos dos de los siguientes hallazgos.


– Circunferencia de la cintura ≥102 cm en hombres y ≥88 cm mujeres – Presión arterial ≥130/85 mmHg, o tratamiento farmacológico específico – Triglicéridos plasmáticos ≥150 mg/dL (≥1,70 mmol/L), o tratamiento farmacológico específico – Colesterol de HDL en plasma <40 mg/dL (<1,0 mmol/L) para hombres y <50 mg/dL (<1,3 mmol/L) para mujeres, o tratamiento farmacológico específico. – Prediabetes (niveles de glucosa en ayunas: 100 a 125 mg/dL [5,6 a 6,9 mmol/L], o 2 horas tras sobrecarga oral: 140 a 199 mg/dL [7,8 a 11,0 mmol/L] o HbA1c: 5,7% a 6,4% [39 a 47 mmol/mol]) – HOMA-IR (modelo homeostático para evaluar la resistencia a la i nsulina) ≥2,5 – Nivel de proteína C reactiva de alta sensibilidad en plasma>2 mg/L

Actualmente, la MAFLD se ha convertido en la primera causa de hepatopatía crónica en los países occidentales, presentando una prevalencia en torno al 24% en la población general [3]. La MAFLD presenta una fuerte asociación con los factores que constituyen el síndrome metabólico, aumentando considerablemente la prevalencia en este grupo de sujetos. Se ha estimado una prevalencia de esteatohepatitis entre el 3 y 5% de la población general, la mayoría con alguna comorbilidad metabólica como la diabetes y obesidad [4]. De estos pacientes, aproximadamente el 25% desarrollarán cirrosis y/o carcinoma hepatocelular (CHC).

En general, los pacientes con MAFLD tienen una mortalidad mayor a largo plazo que la población general, siendo su principal causa de muerte la enfermedad cardiovascular seguida de diferentes tipos de cáncer y la propia enfermedad hepática. Dada la morbi-mortalidad hepática y cardiovascular que genera la MAFLD, es importante la identificación temprana y sencilla de estos sujetos para un manejo adecuado y tratamiento que logre disminuir la mortalidad para todas las causas.

**Table:** 


**Los términos relacionados con la esteatosis no alcohólica deben ser reemplazados por los de esteatosis asociada a disfunción metabólica, ya que refleja mejor las actuales concepciones sobre el proceso patológico y permite una mejor clasificación y manejo de los pacientes.**

## Histopatología e historia natural

La MAFLD se subdivide en dos subtipos histológicos: a) esteatosis simple, que incluye pacientes con esteatosis hepática con o sin inflamación leve; y b) esteatohepatitis, caracterizada por la presencia de inflamación y daño hepatocitario (balonización) con o sin fibrosis [5], [6]. Por examen anatomopatológico, el hígado graso incluye: 1) la esteatosis simple, 2) la esteatosis con inflamación lobulillar o portal sin degeneración balonizante, o 3) la esteatosis con degeneración balonizante pero sin inflamación. El diagnóstico de la esteatohepatitis requiere la presencia conjunta de esteatosis, degeneración balonizante e inflamación lobulillar. La inflamación crónica del hígado puede dar lugar a un proceso progresivo de fibrosis que eventualmente y al cabo del tiempo puede desembocar en cirrosis.

**Figura 1:**
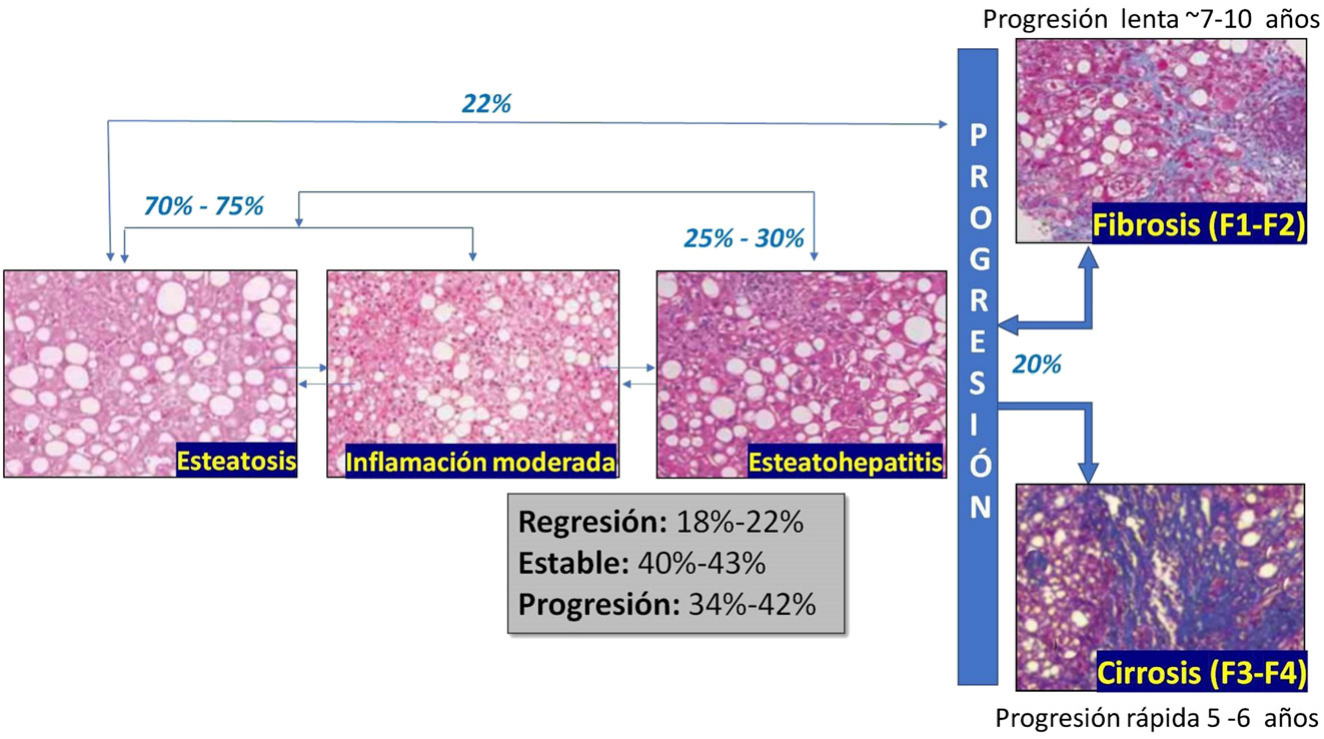
Historia natural de la enfermedad hepática grasa asociada a disfunción metabólica.

La Figura 1 muestra un esquema de la historia natural del MAFLD. Aunque los mecanismos que conducen al desarrollo y progresión de la MAFLD no son completamente conocidos, está ampliamente aceptado que los eventos iniciales son dependientes del desarrollo de obesidad y resistencia a la insulina [7]. Sin embargo, no todos los individuos con MAFLD presentan resistencia a la insulina u obesidad, por lo que es evidente que tanto factores ambientales como genéticos contribuyen a la etiopatogenia de la MAFLD [8]. Adicionalmente, es importante destacar que la regresión de fibrosis en pacientes con MAFLD ha sido descrita, con porcentajes reportados entre un 15 y un 33%, según las particularidades del grupo estudiado [9].

Concretamente, se ha calculado que, en el 7% de los casos, la esteatohepatitis progresa a hepatocarcinoma en un período de 6,5 años. En el 11% de los casos, la esteatohepatitis progresa a cirrosis en un periodo de 15 años [10]. De hecho, la presencia de cirrosis por esteatohepatitis entre los pacientes en lista de espera de trasplante hepático se ha triplicado en la última década, constituyendo hoy en día la segunda causa de inclusión en lista en Estados Unidos [11].

El principal factor predictor evolutivo y de mortalidad de la MAFLD es la presencia de fibrosis hepática, ya que la fibrosis determina el riesgo de desarrollo de cirrosis, descompensación hepática, y/o desarrollo de CHC, es decir, determina el riesgo de mortalidad hepática. También se ha visto que el grado de fibrosis se asocia independientemente a la mortalidad por todas las causas, incluida la cardiovascular, muy especialmente en el caso de la esteatohepatitis [12], [13].

Para la MAFLD el sistema histológico de estadificación de la fibrosis más empleado es el descrito por Brunt et al. [14], donde F1 es definida como fibrosis perisinusoidal o periportal, F2 como fibrosis perisinusoidal con extensión portal o periportal, F3 como fibrosis en puentes y, F4 como cirrosis. El término de fibrosis significativa se refiere a un estadio F2 o superior, y fibrosis avanzada a un estadio F3 o superior. La biopsia hepática es el método de elección para diagnosticar de forma certera MAFLD, y para distinguir entre esteatosis simple y esteatohepatitis, permitiendo clasificar la enfermedad según el grado de actividad (inflamación y daño celular) y el estadio de fibrosis. Pero la biopsia, además de ser un método invasivo, con riesgo potencial de complicaciones, presenta también una serie de limitaciones que se deben fundamentalmente a fallos en el muestreo y a la variabilidad inter e intra-observador. Por este motivo, ha existido interés en el desarrollo de métodos no invasivos de diagnóstico de esteatohepatitis y fibrosis como herramientas de primera línea, que ayuden a identificar aquellos sujetos con una enfermedad hepática significativa y mayor riesgo de mortalidad. Entre estos métodos no invasivos se encuentran los marcadores séricos basados en parámetros analíticos, como veremos más adelante.

**Table:** 


**Establecer la presencia y el grado de fibrosis en la MAFLD es importante, ya que este parámetro se asocia a la mortalidad por todas las causas.**

## Perfil analítico y diagnóstico

La mayoría de los pacientes con MAFLD están asintomáticos y la sospecha de la esteatosis viene dada por un hallazgo casual analítico, dentro de un examen de salud, de una alteración del perfil hepático; o bien por una alteración en la morfología o en la ecogenicidad hepática detectada por un estudio de imagen realizado por otro motivo [15], [16].

Analíticamente, los pacientes con MAFLD pueden presentar un incremento en los valores de concentración de aminotransferasas, siendo la MAFLD la causa principal de una elevación persistente de estas enzimas hepáticas. Sin embargo, valores normales de aminotransferasas no excluyen la presencia de MAFLD; de hecho, la mayoría de estos pacientes presentan unas aminotransferasas consideradas normales [17], [18]. Cuando están elevadas, el aumento de concentración de ambas enzimas, alanino aminotransferasa (ALT) y aspartato aminotransferasa (AST), es ligeramente superior al límite superior del intervalo de referencia. La relación AST/ALT suele ser inferior a 1 en estadios iniciales de la esteatosis y, si esta relación se invierte, puede ser signo de evolución a fibrosis. Sin embargo, el grado de elevación de las aminotransferasas no se relaciona con el grado de fibrosis o inflamación hepática. De forma similar a las aminotransferasas, la gamma-glutamiltransferasa (GGT) también puede encontrarse elevada de forma frecuente en pacientes con EHNA, y su elevación ha sido relacionada con riesgo de fibrosis [19]. Por su parte, la fosfatasa alcalina también puede estar ligeramente elevada, aunque raramente es la única magnitud hepática alterada.

Otro hallazgo analítico que se observa con frecuencia es la elevación de la concentración sérica de ferritina y del índice de saturación de la transferrina, sin que se haya demostrado un aumento paralelo en los depósitos hepáticos de hierro [20]. Algo similar sucede con la presencia de títulos séricos elevados de autoanticuerpos, que aparece con cierta frecuencia en la MAFLD y se consideran un epifenómeno [21], aunque hay estudios que les atribuyen una implicación pronóstica a la enfermedad hepática [22], [23]. La bilirrubina y la albumina no suelen alterarse, excepto en los pacientes con cirrosis, que también pueden presentar aumento del tiempo de protrombina, trombocitopenia y neutropenia.

En la práctica clínica habitual, la ecografía es la prueba de imagen de primer nivel en caso de sospechar una MAFLD debido a su amplia disponibilidad, bajo coste y seguridad; sin embargo, su principal limitación es que presenta una sensibilidad limitada para la detección de esteatosis leve, no detecta esteatosis si esta es inferior al 20% o en individuos con IMC>40 [24], sin mencionar que no es capaz de diferenciar entre esteatosis simple y esteatohepatitis.

Un elemento clave en el diagnóstico de MAFLD es la diferenciación de esteatosis simple y esteatohepatitis y la estadificación de la fibrosis hepática, ya que los pacientes con esteatohepatitis y fibrosis son los que mayor riesgo tienen de desarrollar complicaciones hepáticas y enfermedad cardiovascular. Como ya mencionamos antes, el método de elección para evaluar el grado de lesión histológica sigue siendo la biopsia hepática pero hay que recordar que esta es una prueba invasiva, no exenta de complicaciones y que sus características sumadas a la elevada prevalencia de la EHNA dificulta su realización en la mayoría de los pacientes, restringiéndose su uso a algunas situaciones concretas. Así, solo se recomienda considerar la realización de biopsia hepática en aquellos casos de dudas en cuanto a la presencia de fibrosis, gravedad de la enfermedad o coexistencia de otras enfermedades hepáticas y/o en los pacientes con mayor riesgo de presentar esteatohepatitis y/o fibrosis avanzada [5], [25], [26], que pueden ser identificados previamente de forma más accesible. De ahí la utilidad de los métodos no invasivos de diagnóstico y evaluación de MAFLD que ayudan a caracterizar a los pacientes, determinar su riesgo de progresión de la afectación hepática [16], y evitar la biopsia hepática en los casos no imprescindibles.

Los métodos no invasivos pueden ser divididos en dos grandes grupos: los marcadores “biológicos” o séricos o y los marcadores “físicos” o radiológicos. Entre estos últimos se encuentran la elastografía de transición (FibroScan^®^) y la elastografía por Resonancia Magnética. Los marcadores séricos bioquímicos están tomando gran auge gracias la validez, reproducibilidad y sencillez en su realización, lo que permitiría establecerlos como una herramienta de primera línea tanto en Atención Primaria como en Especializada para la identificación de pacientes que requieren estudios adicionales. A continuación, hablaremos de los diferentes marcadores séricos descritos para la detección de los tres componentes histológicos característicos de la MAFLD (esteatosis, inflamación y fibrosis).

**Table:** 


• **La inclusión de la GGT en el perfil hepático básico puede ayudar a esclarecer el diagnóstico y pronóstico de la MAFLD.**
**• En el manejo de la MAFLD es útil evaluar con métodos no invasivos la probabilidad de una lesión histológica importante que justifique o no la realización de una biopsia hepática.**

## Índices séricos de esteatosis

Se han desarrollado varios índices serológicos para predecir la existencia de esteatosis hepática: Fatty liver index (FLI) [27], SteatoTest [28]**,** NAFLD-Liver Fat Score [29] y Hepatic Steatosis Index (HSI) [30]. En la Tabla 2 se encuentra un resumen de las variables que constituyen cada uno. Estos índices han sido validados tanto en población general como en población con obesidad; se asocian a la resistencia a la insulina y predicen de manera variable futuros eventos metabólicos, hepáticos y cardiovasculares. Indican de manera fiable la presencia de esteatosis pero no permiten cuantificar el contenido de grasa hepática [31]**.**

**Tabla 2:** Índices bioquímicos para la predicción de esteatosis hepática.

	Marcadores bioquímicos	Otras variables	Ref.
Fatty liver index (FLI)	Triglicéridos, GGT	IMC, Cintura (cm)	[27]
Steatotest^a^	*α*2-macroglobulina, haptoglobina, apo A1, bilirrubina, GGT, ALT, glucosa, triglicéridos, colesterol	Edad, sexo, IMC	[28]
NAFLD –Liver fat score	Insulina, AST, ALT	DM, síndrome metabólico	[29]
Hepatic steatosis index (HIS)	ALT, AST	IMC, DM	[30]

^a^Índice patentado. Apo A1, apolipoproteína A1; IMC, índice de masa corporal; DM, diabetes mellitus.

Entre estos los más extendidos y mejor validados están el FLI y el HSI. Valores de FLI por debajo de 30 permiten descartar la presencia de esteatosis con una razón de verosimilitud (likelihood ratio) de 0,2; valores de FLI mayores o iguales que 60 permiten inferir la presencia de esteatosis con una razón de verosimilitud de 4,3. Por su parte, un HSI de menos de 30 indica que se puede descartar MAFLD (con un índice de probabilidad negativo de hasta 0,186) mientras que un HSI de 36 o más indica que el MAFLD está presente (con un índice de probabilidad positivo a partir de 6,069)

Estas puntuaciones pueden estar influidas por la presencia de inflamación hepática y fibrosis, y dado que no aportan grandes ventajas frente a las técnicas de imagen y la analítica de rutina, su empleo en la práctica clínica habitual no está extendida [16]. Sin embargo, su amplia disponibilidad, su bajo coste y su accesibilidad desde la atención primaria las convierten en una alternativa razonable como primer paso de cribado ante la sospecha de MAFLD. Algunos autores abogan por incluir al FLI como un primer paso en el cribado de enfermedad hepática avanzada en población general, al seleccionar primeramente a aquellos sujetos con esteatosis [32].

**Table:** 


• **Por su disponibilidad, los índices séricos de esteatosis deben ser la primera opción de cribado de esteatosis en población susceptible.**
• **Los índices FLI y HSI serían la mejor opción, ya que son los mejor validados en nuestro entorno.**

## Marcadores séricos de esteatohepatitis

Los marcadores séricos evaluados para predecir la existencia de esteatohepatitis están relacionados con las vías fisiopatológicas de la enfermedad (apoptosis / muerte celular, inflamación y estrés oxidativo). El más estudiado es la citoqueratina 18 fragmentada (CK18-F), un producto de degradación de la apoptosis de hepatocitos [33]. Un metaanálisis determinó para este producto un AUROC de 0,82 y una sensibilidad y especificidad del 75 y 77% respectivamente para predecir esteatohepatitis, es decir, una precisión diagnóstica limitada; además los valores de corte publicados son muy variables [34]. No obstante, al incluir este marcador en diversos paneles la eficacia diagnóstica puede aumentar hasta un 0,92 [35] por lo que se necesitan futuros estudios para determinar el alcance de la utilidad de este marcador.

Otros marcadores estudiados son varias hormonas como el *fibrobast growth factor 21* (FGF21) y la adiponectina, pero con muy baja precisión diagnóstica. También se han estudiado marcadores de estrés oxidativo y de inflamación como la interleucina 6 y el *tumor necrosis factor-α* (TNFα). Todos ellos han sido evaluados en series cortas o estudios piloto en grupos heterogéneos de pacientes con resultados contradictorios, y ninguno es capaz de diferenciar NASH de esteatosis simple con alta sensibilidad y especificidad [36]. Con el fin de mejorar la precisión diagnóstica de los marcadores se han desarrollado modelos predictivos que combinan alguno de estos biomarcadores séricos con parámetros analíticos y variables clínicas, incluso polimorfismos genéticos, pero no han sido validados adecuadamente por lo que, de momento, no son recomendables en la práctica clínica [37].

Los estudios basados en la metabolómica han permitido el desarrollo por un grupo español, de un test (OWL Liver Test) que permite diferenciar la esteatohepatitis de la esteatosis simple con una buena sensibilidad y especificidad (ROC superior a 0,8); este test se ha obtenido mediante el análisis de muestras de 465 pacientes [38] y ha sido validado en estudios ciegos sobre 2 cohortes independientes [39]. Las limitaciones de uso en otras etnias y en pacientes diabéticos no controlados se ha resuelto recientemente incorporando las transaminasas y la hemoglobina glicosilada (HbA1c) al algoritmo diagnóstico previo [40]. El test está validado y comercializado con marcado CE. Recientemente, desde la Sociedad española de patología digestiva (SEPD) se ha puesto en marcha un estudio multicéntrico, denominado “NASH Registry” que tiene, entre otros fines, el evaluar la utilidad de la implementación de esta herramienta.

**Table:** 


**Son necesarios más estudios para validar el impacto en la práctica clínica de los marcadores séricos de esteatohepatitis.**

## Marcadores séricos de fibrosis hepática

Toda enfermedad hepática crónica se caracteriza por inflamación, balonización, necrosis o apoptosis hepatocitaria y fibrosis, pero en numerosos estudios se ha visto que es la fibrosis el factor que más determina la progresión de la enfermedad hepática [12], [41], [42], [43], por este motivo ante un paciente con hepatopatía crónica es obligado determinar su grado de fibrosis. Además, especialmente en el caso de la MAFLD, el grado de fibrosis se ha asociado de forma independiente al riesgo cardiovascular y a la mortalidad por todas las causas [12], [13].

Para el clínico no es complicado diagnosticar a un paciente de cirrosis hepática cuando ya ha presentado alguna descompensación de su hepatopatía (ascitis, hemorragia digestiva por varices esofágicas, encefalopatía hepática). Sin embargo, es en las fases más precoces (fibrosis F2 o fibrosis significativa) cuando más se puede modificar el pronóstico de un paciente. Dado que por norma general es una patología asintomática, para conseguir diagnosticar al paciente en estas fases adquieren gran relevancia los marcadores séricos.

Los marcadores séricos de fibrosis hepática se pueden clasificar en: 1) marcadores indirectos, relacionados con la función hepática como la albúmina, bilirrubina, AST y ALT, y; 2) marcadores directos, que son componentes de la matriz extracelular como el ácido hialurónico, metaloproteinasas de matriz y los subtipos de colágeno [44], [45]. Numerosos de estos marcadores se han evaluado en pacientes con MAFLD, sin que ninguno de ellos alcance una eficiencia diagnóstica deseable. Por tanto, lo que se ha establecido son modelos predictivos que combinan dichos marcadores séricos con características clínicas del paciente [46], [47], [48], [49], [50], [51], [52], [53], [54], [55], [56], [57], [58], [59], [60], [61], [62], [63], [64], [65], [66], [67], [68], y que están resumidos en la Tabla 3.

**Tabla 3:** Índices para la valoración no invasiva de fibrosis hepática, mostrando las variables que los componen clasificadas en marcadores bioquímicos indirectos, directos y otras variables.

	Marcadores bioquímicos indirectos	Marcadores bioquímicos directos	Otras variables	Ref.
NAFLD Fibrosis Score (NFS)	Glucosa, plaquetas, AST, ALT, albúmina	–	Edad, IMC	[46], [47]
Fibrosis-4 (FIB-4)	Plaquetas, AST, ALT	–	Edad	[58], [62]
Enhanced Liver Fibrosis (ELF) ^a^		Ácido hialurónico, PIIINP, TIMP-1		[64], [65]
BARD score	AST, ALT		IMC, DM	[66]
AST to Platelet Ratio Index (APRI)	AST, plaquetas			[48], [68]
FibroTest ^a^	*α*2-macroglobulina, haptoglobina, apo A1, GGT, bilirrubina		Edad, sexo	[49]
ActiTest	*α*2-macroglobulina, haptoglobina, apoA1, GGT, bilirrubina, ALT			[50]
Hepascore^a^	Bilirrubina, GGT, *α*2-macroglobulina	Ácido hialurónico	Edad y sexo	[51], [52]
PGA	Índice de protrombina, GGT, apo A1			[53]
FibroIndex	Plaquetas, AST, *Ύ*-globulina			[54]
Forns	GGT, colesterol, plaquetas		Edad	[55]
Fibrometer NAFLD	Glucosa, AST, ALT, ferritina, plaquetas		Edad, peso	[48], [56]
FibroSpect II ^a^	*α*2-macroglobulina	Ácido hialurónico, TIMP-1		[57]
SHASTA	AST, albúmina	Ácido hialurónico,		[59]
Hepamet Fibrosis Score (HFS)	AST, albúmina, plaquetas, HOMA (glucosa e insulina)		Edad, Sexo, DM	[60]
ADAPT	Plaquetas	PRO-C3	Edad, DM	[61]

^a^ Índices patentados. Apo A1, apolipoproteína A1; IMC, índice de masa corporal; DM, diabetes mellitus; PIIINP, propéptido aminoterminal del procolágeno tipo III; TIMP-1, inhibidor tisular de metaloproteinasas tipo 1.

Entre los modelos predictivos de fibrosis avanzada se distinguen dos grandes grupos: a) los modelos simples, que usan una combinación de parámetros analíticos de rutina con variables clínicas, y; b) los modelos complejos, que usan marcadores séricos relacionados con el proceso y degradación de matriz extracelular (directos). Es importante tener en cuenta que los marcadores incluidos en estos índices de fibrosis pueden verse alterados por otras causas, como la trombocitopenia debida a causas no relacionadas con patología hepática, los aumentos de GGT inducidos por los tratamientos antirretrovirales, alcohol o fármacos con toxicidad hepática, el síndrome de Gilbert, la hemólisis y la colestasis, entre otros [25], [56], [69], [70]**.** En cuanto a los marcadores directos, éstos tienden a reflejar mejor la tasa de recambio de matriz extracelular que la cantidad de matriz depositada, por lo que las concentraciones suelen ser mayores cuando el grado de inflamación es mayor y, por el contrario, una deposición de matriz elevada puede infravalorarse si la inflamación es mínima. Por otro lado, ninguno de los marcadores es específico de hígado y pueden alterarse en presencia de otras enfermedades y estados (postoperatorio) asociados a fibrosis, por lo que la presencia de inflamación extrahepática concurrente puede contribuir al aumento de las concentraciones séricas del marcador. Adicionalmente, la liberación de algunos marcadores puede verse afectada por la disfunción de las células endoteliales sinusoidales o la disminución de la excreción biliar de la propia enfermedad hepática. De cualquier forma, y en especial para los modelos simples, dada su accesibilidad, bajo coste y elevado valor predictivo negativo, hacen recomendable su uso como aproximación diagnóstica inicial, sin olvidar hacer una interpretación crítica de sus resultados. En la Tabla 4 se muestra el valor diagnóstico en los puntos de corte alto de los principales índices serológicos de fibrosis avanzada en pacientes con MAFLD.

**Tabla 4:** Valor diagnóstico en los puntos de corte alto de los principales índices serológicos de fibrosis avanzada en pacientes con MAFLD.

	Cut-off	AUROC	Sens, %	Esp, %	VPP, %	VPN, %	Ref
NFS	≥0,676	0,84	43	96	82	80	[66]
ELF	9,8	0,90	80	90	71	94	[65]
FIB-4	≥3,25	0,80	26	98	75	85	[63]
APRI	≥1	0,77	43	86	34	90	[67]
HFS	≥0,47	0,87	35	97	72	82	[60]

AUROC, área bajo la curva ROC; Sens, sensibilidad; Esp, especificidad; VPP, valor predictivo positivo; VPN, valor predictivo negativo; NFS, NAFLD fibrosis score; ELF, enhanced liver fibrosis; HFS, hepamet fibrosis score.

Dentro de estos modelos predictivos de fibrosis, dos son los más estudiados y validados en diferentes poblaciones de pacientes con MAFLD, fibrosis-4 (FIB-4) y NAFLD Fibrosis Score (NFS). Valores del FIB-4 por debajo de 1,30 permiten excluir la presencia de fibrosis avanzada con un VPN del 95%; mientras que valores por encima de 3,25 indican una fibrosis avanzada con un VPP del 75% [63]. En cuanto al NFS, un estudio multicéntrico con más de 700 pacientes obtuvo un VPN de 88–93% para excluir fibrosis avanzada con valores por debajo de −1,455; y un VPP de 82–90% para valores por encima de 0,676 [71]. No hay que olvidar la influencia de las comorbilidades como la edad avanzada, la diabetes y la obesidad en el resultado de estos índices. En este sentido, Hepamet Fibrosis Score (HFS), un índice recientemente desarrollado en nuestro país y validado, ha demostrado una Odds Ratio diagnóstica significativamente mayor para el punto de corte bajo (<0,12) y el alto (>0,47) que el FIB-4 y NFS para descartar/diagnosticar fibrosis avanzada independientemente de la presencia o ausencia de diabetes, del IMC y de los grupos de edad [60], siendo probablemente el mejor marcador indirecto de fibrosis hepática en este momento.

Otro índice prometedor desarrollado y publicado recientemente es el ADAPT, en cuya fórmula incluye la edad, la presencia de diabetes, las plaquetas y el PRO-C3, un marcador de formación de colágeno III que en varios estudios se ha visto que aumenta con la fibrosis y no sólo en la etiología de MAFLD [61]. Para este índice se ha determinado un AUROC de 0,89 tanto en la cohorte de derivación como en la de validación, siendo esta precisión diagnóstica superior a otros índices como FIB-4 y NFS.

**Table:** 


• **Los índices séricos de fibrosis constituyen una primera aproximación para la exclusión de fibrosis significativa en pacientes con MAFLD.**
• **Tanto el FIB-4 como el NFS están validados en población con MAFLD y tienen una alta eficiencia diagnóstica. El uso del índice HFS es también recomendable.**
• **En los pacientes con resultados de índices séricos sugestivos de fibrosis significativa debe ampliarse su estudio mediante otros métodos bioquímicos, radiológicos y/o biopsia.**
• **En la evaluación crítica de los resultados de estos índices es importante tener en cuenta que los marcadores incluidos pueden verse alterados por causas ajenas a la hepatopatía metabólica.**

## Uso en práctica clínica

Ante todo paciente con sospecha de MAFLD se deberían emplear en práctica clínica pruebas no invasivas para estratificar su riesgo. La elección de esta va a depender especialmente de la disponibilidad local. En el ámbito de la atención primaria, los índices séricos simples de fibrosis con un alto valor predictivo negativo deberían usarse como primera línea dada su sencillez, bajo coste y alta disponibilidad.

Como ya hemos mencionado antes, los marcadores séricos presentan una serie de limitaciones o factores de confusión que deben tenerse en cuenta a la hora de interpretar el resultado.

En cuanto a marcadores radiológicos la elastografía de impulso de fuerza de radiación acústica (ARFI), la elastografía de ondas de corte Shear Wave (SWE) y la elastografía transitoria con medición de parámetros de atenuación controlada (controlled attenuation parameter; CAP) pueden evaluar estatosis y fibrosis simultáneamente [72]. Limitaciones similares a las mencionadas para los marcadores séricos existen también en estos casos. Con el FibroScan^®^ existen limitaciones como la obesidad mórbida o un espacio intercostal estrecho, y su resultado puede verse influido por cualquier proceso que modifique la consistencia hepática como la esteatosis, la congestión venosa por fallo cardíaco, o la inflamación aguda en caso de hepatitis aguda. Con los métodos basados en la resonancia magnética, su principal inconveniente es la escasa disponibilidad y el alto coste. Por este motivo, algunos autores han propuesto estrategias que combinan marcadores séricos con marcadores radiológicos siendo el más empleado de estos últimos el FibroScan [73], [74], [75]. La combinación aumenta la precisión diagnóstica de fibrosis avanzada y disminuye considerablemente la necesidad de realizar biopsia hepática. Pero esta combinación no debería ser simultánea ya que, aunque mejora la sensibilidad y especificidad, aumenta enormemente el área de incertidumbre [75]. No ocurre lo mismo cuando la combinación es secuencial, reduciendo el área de incertidumbre manteniendo una buena sensibilidad y especificidad [76].

El desarrollo de algoritmos diagnósticos de MAFLD secuenciales debe iniciarse con índices séricos ampliamente validados, asequibles y con un elevado valor predictivo negativo como son el FIB-4 o NFS. Pacientes con valores de FIB-4<1,3 o NFS<−1,455 se considera que tienen bajo riesgo de fibrosis avanzada y no necesitan pruebas diagnósticas adicionales, pero aquellos pacientes con riesgo intermedio (FIB-4: ≥1,3 – <3,25 o NFS: ≥−1,455 – <0,676) o riesgo alto de fibrosis avanzada (FIB-4>3,25 o NFS>0,672) han de remitirse a un centro de referencia para una evaluación hepática adicional, ya sea con marcadores radiológicos o séricos directos. La combinación de FIB-4 o NFS con ELF es recomendada por la NICE (The National Institute of Health, UK) para el diagnóstico de la fibrosis avanzada en pacientes con esteatohepatitis [77].

**Figura 2:**
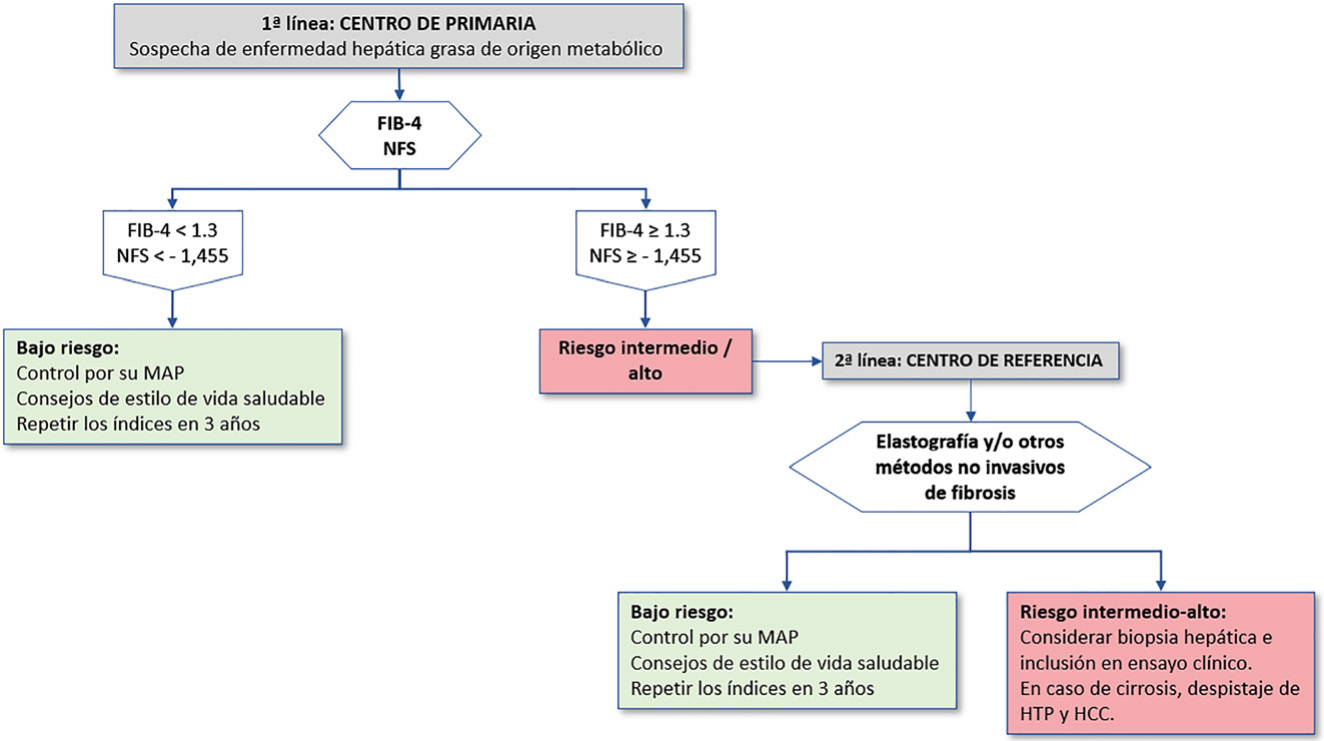
Propuesta de algoritmo para la detección de fibrosis hepática; basado en la estratificación del riesgo mediante marcadores no invasivos en pacientes con sospecha de esteatosis hepática. NFS, NAFLD Fibrosis Score; HTP, hipertensión portal; HCC, hepatocarcinoma.

En la Figura 2 se muestra una propuesta de manejo de la MAFLD fundamentada en la evidencia clínica actual, y que parte de la sospecha de MAFLD basada en la presencia de esteatosis detectada por ecografía o la existencia de factores metabólicos de riesgo (obesidad, diabetes mellitus tipo 2 o síndrome metabólico) [37], [78]**.**

**Table:** 


• **Ante todo paciente con sospecha de MAFLD se deberían emplear las pruebas no invasivas para estratificar su riesgo.**
• **El desarrollo de algoritmos diagnósticos de MAFLD secuenciales debe iniciarse con índices séricos ampliamente validados, asequibles y con un elevado valor predictivo negativo como son el FIB-4, el NFS o el Hepamet.**
• **Los pacientes con resultados de índices séricos no sugestivos de fibrosis significativa no requieren estudios adicionales y pueden ser reevaluados con una nueva medición de índices séricos en un año.**

## Una oportunidad para un diagnóstico precoz de las enfermedades hepáticas

Los servicios de análisis clínicos de nuestros hospitales tienen una oportunidad única para contribuir al control, diagnóstico y estadiaje de la enfermedad hepática más frecuente, la enfermedad hepática grasa asociada a disfunción metabólica. Y esta oportunidad pasa por la incorporación a su cartera de servicios de estos índices de esteatosis y de fibrosis, índices que se pueden automatizar de forma sencilla y contribuirán a la difusión del conocimiento de la enfermedad. Desde las sociedades científicas, no solo de Análisis Clínicos, sino de Digestivo, Medicina Familiar y Comunitaria, Medicina Interna, Endocrinología y otras, se debe insistir en esta necesidad.

## Conclusiones

La MAFLD se ha convertido en la primera causa de hepatopatía crónica en el mundo occidental dada su estrecha relación con la obesidad y el síndrome metabólico. Los principales factores que determinan la progresión de la enfermedad hepática son la presencia de inflamación y, especialmente, fibrosis. Este último factor en los pacientes con MAFLD se ha relacionado no solo con la morbi-mortalidad de causa hepática sino también con el riesgo cardiovascular. Por eso la importancia de evaluar el grado de fibrosis en todo paciente con un diagnóstico de MAFLD. En este contexto, los marcadores séricos nos ayudan a la identificación no invasiva y fácilmente accesible de los pacientes con un potencial riesgo de enfermedad hepática avanzada.
